# Urban Healthy Ageing in Romania: Policy Options for Age-Friendly Cities and Long-Term Care Reform

**DOI:** 10.3389/phrs.2026.1609176

**Published:** 2026-03-24

**Authors:** Sorina Corman

**Affiliations:** Lucian Blaga University of Sibiu, Sibiu, Romania

**Keywords:** digital inclusion, equity, governance, healthy ageing, long-term care

## Abstract

**Background:**

Romania’s rapid population ageing now unfolds primarily in cities, where health, social care, housing and mobility intersect. Within metropolitan areas, older residents face unequal access to community long-term care (LTC), digital services and health-promoting public space.

**Analysis:**

Framed by European Commission and WHO agendas, this brief examines Romania’s national strategies on health, ageing and LTC through an urban lens. It identifies a persistent rhetoric–implementation gap: municipal services remain underfunded and fragmented, and prevention or person-centred models are only weakly embedded in urban planning and budgeting.

**Policy Options:**

Five priorities could align ageing policy with urban health: intersectoral city governance with transparent equity dashboards; legal and financial recognition of informal caregivers; expansion of community hubs integrating primary care, social work and rehabilitation; digital inclusion programmes for older adults; and health-promoting urban design that improves walkability, thermal comfort and access to green/cool spaces.

**Conclusion:**

Converging city governance, LTC reform and urban design can translate policy aspirations into measurable gains in equity, autonomy and healthy life expectancy among older urban residents.

## Background

Romania’s strategic corpus—National Health Strategy 2023–2030, National Strategy for Long-Term Care and Active Ageing 2023–2030, and the Sustainable Development Strategy 2030—appropriates the language of prevention, integration and equity, yet stops short of operational clarity. Responsibilities remain split across ministries and municipal departments; city budgets rarely include measurable ageing targets; and monitoring systems do not routinely track intra-urban inequities in LTC coverage, affordability, digital access or exposure to heat and noise [1–4].

Recent Romanian reform efforts have explicitly acknowledged structural weaknesses in the long-term care system, including institutional fragmentation, insufficient coordination between health and social protection sectors, and the limited development of community-based services. Technical assessments supporting the 2023–2030 LTC Strategy emphasise deinstitutionalisation, workforce professionalisation, and expansion of home-based care [5]. Earlier structural analyses similarly described Romania’s LTC model as highly familialist and under-integrated [6].

Parallel Romanian scholarship has increasingly addressed age-friendly urban environments. Empirical assessments in Bucharest demonstrate significant intra-urban disparities in perceived age-friendliness and neighbourhood accessibility [7]. Additional studies examining smart and age-friendly urban planning in Romania highlight the role of digital infrastructure, spatial analytics and participatory governance in shaping autonomy among older adults [8, 9]. Geospatial analyses published in urban and geomatics journals further map service proximity and environmental exposure as determinants of ageing experiences in Romanian cities [10].

Beyond academia, civic initiatives such as the CONECTATE manifesto advocate for participatory and cross-sectoral ageing policies, while regional developments—including the Active and Healthy Ageing Programme of the Republic of Moldova—frame ageing as a multidimensional governance challenge [11].

However, despite these contributions, Romanian scholarship and policy initiatives remain largely focused on local spatial diagnostics or sectoral reforms. Integration between national LTC governance and urban implementation frameworks remains limited. The present Policy Brief builds upon this emerging literature while examining alignment gaps between national strategy design and municipal operationalisation. According to recent State of Health in the EU profiles, Romania continues to exhibit significant gaps between life expectancy and healthy life expectancy, particularly in urban contexts marked by socio-economic disparities [12].

## Analysis

Romania’s ageing policies are institutionally fragmented, reflecting the country’s broader administrative divide between the health, labour and social-protection sectors. The Ministry of Health oversees preventive and medical services, the Ministry of Labour and Social Solidarity is responsible for social assistance and long-term care (LTC), while municipalities deliver community-level programs within chronically underfunded local budgets. The absence of an interministerial body dedicated to ageing policy has created parallel planning cycles, inconsistent eligibility rules and uneven territorial coverage. For instance, long-term care units under the health system operate separately from social-assistance centres, each reporting to different funding and accountability lines. These governance silos lead to duplication in some areas—such as institutional care—and to complete neglect in others, particularly home-based and respite services [1, 12, 13]. The European Care Strategy explicitly calls for national coordination mechanisms that connect ministries and local authorities, yet Romania has not established such a structure. The resulting institutional fragmentation prevents strategic alignment with European benchmarks on quality, access and workforce development [1, 3, 13].

The fragmentation identified at governance level is mirrored by structural characteristics of the Romanian LTC system documented in earlier analytical work [6]. Recent technical guidance accompanying the 2023–2030 reform agenda reiterates the urgency of clarifying institutional responsibilities and strengthening community provision [5]. Yet municipal implementation capacity remains uneven, particularly in metropolitan areas where demographic ageing intersects with spatial inequality.

Empirical research conducted in Bucharest further confirms that perceived age-friendliness varies significantly according to neighbourhood infrastructure and socio-economic gradients [7]. These findings reinforce the argument that LTC reform cannot be addressed independently of spatial planning, transport accessibility and environmental quality.

Financially, Romania invests less than 0.5% of GDP in LTC—among the lowest in the OECD [3, 4]. Comparative OECD data further confirm Romania’s position in the lower tier of long-term care expenditure *per capita* and workforce density [14, 15]. Cross-national evidence also shows that systems with stronger community-based care provision demonstrate lower rates of avoidable institutionalisation [16]. Most allocations are absorbed by hospital or residential institutions, leaving minimal investment in community or preventive services. Consequently, many older adults depend on unpaid family members for daily care, especially in rural and peri-urban areas where formal services are scarce. This familialist model, while culturally familiar, produces gendered inequalities and economic strain. Women, often daughters or spouses, withdraw from the labour market to provide full-time care without formal recognition, training or pension credits [10]. European evidence demonstrates that such informal care, when unsupported, leads to caregiver burnout and worsened outcomes for both caregiver and recipient [13, 17]. A sustainable system thus requires both formalisation and redistribution of resources toward community-based alternatives that value caregiving as social infrastructure rather than private obligation.

Beyond financial or structural considerations, relational and psychosocial dimensions of care shape quality and wellbeing. A scoping review in *Public Health Reviews* underscores how the emotional reciprocity between caregivers and recipients influences not only satisfaction but also cognitive resilience and recovery trajectories [17]. These findings challenge the mechanistic view of LTC as a technical service and re-position it as a relational process requiring empathy, continuity and skill. In Romania, professional caregivers frequently rotate across institutions with limited training in gerontology or communication, while informal caregivers receive almost no guidance. The result is episodic, task-centred care that neglects social connection. The absence of structured community spaces for intergenerational contact or peer support compounds isolation among older residents. Conversely, European experiences show that community centres combining health promotion, cultural participation and light physical activity can delay functional decline and enhance subjective wellbeing [18, 19]. For Romanian cities, integrating such “social prescriptions” into municipal public-health planning would represent a cost-effective preventive strategy consistent with WHO’s person-centred framework [19, 20].

The built environment is an equally important determinant of healthy ageing. Recent PHR reviews on neighbourhood resources and cognitive health highlight that green exposure, noise control and safe walkability directly influence mental performance and functional independence in older adults [21]. In Romania, urban expansion has often prioritised motor traffic and commercial development over public-space accessibility. Sidewalk discontinuities, air pollution and insufficient seating discourage outdoor mobility, while the scarcity of green and cool spaces increases vulnerability to heat waves. The 2023 heat episodes in Bucharest and Iaşi, where older adults mortality spikes mirrored ambient-temperature peaks, exemplify how climate and health intersect at the city scale. Yet local governments rarely incorporate health impact assessments into spatial-planning decisions. Integrating age-friendly design—continuous pavements, shaded routes, accessible benches and barrier-free public transport—would not only improve mobility but also reduce social isolation and healthcare costs. Such environmental interventions are fully aligned with the *WHO Global Age-Friendly Cities Guide* and the *Decade of Healthy Ageing*’s call for environments that enable rather than constrain function [19, 22].

Digitalisation is repeatedly portrayed in policy documents as a vehicle for modernisation and efficiency, yet its implementation reveals a pronounced digital divide. According to Eurostat, only 28% of Romanians possess at least basic digital skills, and among people aged 65+, the rate is below 15% [23, 24]. This technological gap translates into health inequities: many older urban residents cannot navigate online appointment systems, telemedicine platforms or digital reimbursement procedures. The risk of exclusion is particularly acute for those living alone or with sensory impairments. Research in *Nature Medicine* and *Public Health Reviews* demonstrates that digital health can enhance monitoring and continuity of care only when accompanied by inclusive design, assisted access and digital-literacy training [25–28]. In Romania, pilot telemedicine projects exist but remain small-scale and lack evaluation. Municipal partnerships with universities, NGOs and libraries could create digital-inclusion programmes offering basic training, device loans and guided access to online health portals. Such initiatives would transform digitalisation from an administrative reform into a social-equity tool consistent with EU digital-skills targets for 2030 [23, 24].

Taken together, these findings portray a system rhetorically committed to European principles but constrained by institutional inertia, fiscal scarcity and limited cross-sector collaboration. The core challenge is not conceptual innovation but operational integration—translating existing strategies into accountable, adequately funded and locally adaptable frameworks. By linking governance reform, workforce support, digital inclusion and health-promoting urban design, Romania could gradually shift from reactive, institution-centred policies to preventive, person-centred and community-anchored models of healthy ageing. These structural and spatial governance gaps are summarised in Table 1, which provides an integrated overview of the principal implementation challenges and their corresponding policy options.

**TABLE 1:** Key governance gaps and corresponding policy responses (Romania, 2024).

Identified gap	Consequence	Proposed policy response
Institutional fragmentation	Uneven service coverage	Intersectoral urban ageing council
Underfunded community care	Overreliance on institutional LTC	Reallocation toward home-based services
Informal caregiver precarity	Gender inequality	Legal recognition and pension credits
Environmental barriers	Reduced mobility	Age-friendly urban design standards
Digital divide	Service exclusion	Municipal digital literacy programs

## Policy Options

Addressing Romania’s urban ageing challenges requires more than marginal program adjustments; it calls for a systemic reframing of how health, care, and the urban environment are governed. International experience demonstrates that countries able to bridge the health–social divide have relied on durable institutional mechanisms that cut across sectors and levels of administration [3, 13, 19]. For Romania, the starting point should be the establishment of a permanent Urban Ageing and Long-Term Care Council, mandated to coordinate national ministries, county councils, and municipalities. This body would serve as both a planning forum and an accountability mechanism, ensuring that national strategies translate into measurable city-level actions. Its main task would be to develop equity dashboards—annual, publicly accessible reports tracking indicators such as LTC service coverage by district, affordability of home-based care, density of green and cool public spaces, and accessibility of digital infrastructure [3, 5, 13, 19]. Publishing such metrics would not only enhance transparency but also make disparities visible, guiding funding toward high-need areas and aligning local performance with European Care Strategy benchmarks.

Equally vital is the recognition and professionalisation of caregiving. Romania remains one of the few EU countries where informal carers have no legal status or financial protection [1, 13]. In practice, care is sustained by family members—mostly women—who interrupt their employment to provide assistance without remuneration or pension rights [10]. This arrangement generates gender inequalities, hidden poverty and loss of human capital. A coherent response should include legal recognition of caregivers, direct financial support, access to training, and integration into social insurance systems. Such reforms would not only acknowledge care as socially valuable labour but also create a bridge between formal and informal provision. Evidence from comparative European research shows that where carers are supported—through respite services, peer networks and income replacement—older people remain independent for longer and health outcomes improve [13, 17, 19]. In the urban context, municipalities could pilot caregiver support centres, co-located with community health hubs, to provide psychological counselling, legal advice, and training modules on geriatric care and digital tools.

Rebalancing the system toward community-based and preventive services is another central policy direction. Romania’s current long-term care landscape remains dominated by institutional and hospital-based provision, absorbing the bulk of public funds while reaching a limited number of beneficiaries [3, 4]. International comparative analyses of OECD countries suggest that integrated LTC systems combining health and social funding streams are associated with improved access and continuity of care [29]. Typologies of long-term care regimes further illustrate how fragmented, residual systems struggle to transition toward preventive and community-oriented models [30]. International evidence, including systematic reviews published in Public Health Reviews, shows that community-oriented interventions—especially those combining health promotion, social participation and rehabilitation—yield better functional outcomes and higher satisfaction at lower cost [18, 19, 21]. Municipalities could therefore transform underused facilities into integrated community hubs that deliver primary care, social work, physiotherapy and cultural or physical-activity programs for older adults. Such hubs, developed through partnerships between local authorities, NGOs and universities, would provide a physical anchor for healthy ageing at neighbourhood level, particularly in dense urban districts where isolation and loneliness are acute.

Beyond service delivery, urban planning itself must become a vector of health policy. The WHO Global Age-Friendly Cities Guide and the Decade of Healthy Ageing framework both emphasise the role of built environments that enable function and participation [19, 22]. Romanian municipalities often approach planning as a technical exercise centred on land use and infrastructure rather than wellbeing. Yet environmental determinants—walkability, green access, thermal comfort and noise control—shape morbidity and mortality as profoundly as medical factors. Integrating health impact assessments into urban planning decisions would make health objectives explicit in zoning, housing and transport policy. Cities could establish minimum standards for shaded pedestrian routes, accessible benches, safe crossings and barrier-free public transport. Embedding health objectives into urban governance aligns with the Healthy Cities framework and recent WHO guidance on urban health governance, which emphasise intersectoral coordination, participatory planning, cross-sector accountability mechanisms, and equity-oriented monitoring systems [31]. Experience from European “superblock” and “15-min city” initiatives demonstrates that urban design promoting proximity and mobility not only benefits older adults but also reduces pollution and fosters intergenerational interaction [19, 21, 22]. For Romania, embedding these principles in municipal building codes and budget allocations would operationalise the “health in all policies” paradigm.

Another transformative pathway lies in digital inclusion. The digital divide is now a social determinant of health: exclusion from online systems can mean exclusion from services themselves. According to Eurostat, fewer than one in six Romanians aged 65+ possess basic digital skills, and the gap between urban and rural areas exceeds 30 percentage points [23, 24]. Bridging this divide requires policies that go beyond infrastructure toward empowerment. Municipalities could implement lifelong-learning initiatives offering tailored training for older adults in libraries, cultural centres or primary-care facilities. Such programs should combine technical guidance with accessible device loans and one-on-one assistance. International evaluations show that digital-literacy training increases healthcare access, social participation and self-efficacy among seniors [25–28]. In parallel, e-health platforms must be designed according to universal-access principles—clear language, voice assistance, adjustable fonts and offline functionality—to avoid reproducing exclusion within digital environments. Romania’s national digital strategy could thus evolve from a technology procurement plan into a genuine social innovation policy supporting ageing in place.

Financing and incentives are necessary to sustain these reforms. Redirecting even a small portion of institutional-care expenditure toward community-based prevention would generate multiplier effects across health and social sectors. For instance, OECD simulations suggest that reallocating 0.2% of GDP from hospital care to home-based services could expand access by 30% and reduce hospital readmissions by 10% [3, 4]. European funds available under cohesion and recovery programs provide a unique window for such rebalancing. To ensure continuity after external financing ends, Romania should establish a dedicated National Long-Term Care Fund, pooling contributions from state, local and social-insurance budgets. Allocation criteria could prioritise municipalities demonstrating progress on equity indicators and integration outcomes, thereby linking funding to performance rather than historical spending patterns.

Finally, monitoring and evaluation must become integral to the policy cycle. Romanian strategies often include broad goals but few measurable targets. Developing a national indicator framework aligned with WHO and EU standards would make progress visible and comparable. Indicators could include coverage rates of home-based care, average waiting times for community services, caregiver training completion, and proportion of older adults participating in digital-literacy programs. Regular publication of these metrics would institutionalise accountability and inform citizens, researchers and policymakers alike.

In sum, the transition toward healthy urban ageing is not a single reform but a continuum of policy shifts—governance integration, workforce recognition, community investment, environmental design and digital empowerment. Implemented together, these measures can transform Romanian cities from reactive service providers into proactive enablers of wellbeing, echoing the vision articulated by the European Care Strategy and the Decade of Healthy Ageing [13, 19]. The challenge is not lack of vision but of sustained, evidence-based implementation—an area where Public Health Reviews and comparative research can continue to guide national and local actors in building cities that support dignity, participation and resilience across the life course.

## Conclusion

Romania’s experience with ageing policy exemplifies the tension between rhetorical convergence with European frameworks and the realities of fragmented implementation. Over the past decade, successive strategies have echoed the vocabulary of the European Care Strategy and the Decade of Healthy Ageing, invoking concepts such as integration, prevention, and equity. Yet these remain largely aspirational in the absence of coherent governance and sustainable financing. The country’s ageing policy thus mirrors a broader regional pattern in which demographic change outpaces institutional adaptation. In Romania’s case, the challenge is magnified by urban inequality: metropolitan centres accumulate both opportunities and vulnerabilities, while municipal authorities lack the mandate or resources to coordinate health, social and spatial interventions.

This implementation gap echoes broader global monitoring findings, which underline uneven progress across countries during the first years of the Decade of Healthy Ageing [32].

Bridging this policy–practice divide requires an explicit recognition that ageing is not solely a social or medical issue but a structural determinant of urban wellbeing. Cities are the physical and administrative spaces where health, housing, transport, and digital access intersect; therefore, they must become the laboratories for healthy-ageing innovation. Governance reform at national level—through an intersectoral council for ageing and long-term care—should be complemented by empowered municipalities equipped with data systems, equity dashboards and performance-based funding. In practice, this would mean measurable commitments: expanding home and community care, supporting caregivers, investing in digital literacy, and embedding health impact assessments into urban planning. The cumulative effect would be a more coherent, person-centred system that treats older adults not as dependents but as citizens with rights and capabilities.

This Policy Brief has several limitations. First, it relies on secondary policy documents and publicly available data rather than primary empirical fieldwork. Second, subnational disparities are discussed conceptually rather than through original quantitative modelling. Third, the rapid evolution of digital and long-term care reforms may render certain implementation gaps time-sensitive. Nevertheless, the strength of the analysis lies in its integrative perspective, linking governance, urban planning, workforce sustainability and digital inclusion within a coherent healthy-ageing framework grounded in European and WHO guidance.

From a broader European perspective, Romania’s trajectory holds lessons for other middle-income countries undergoing similar transitions. The evidence reviewed in *Public Health Reviews*—from relational care and social engagement to environmental and digital determinants—demonstrates that sustainable ageing is a function of integration rather than innovation alone. Building age-friendly cities entails aligning physical space, service design and governance with the lived realities of older people. By translating policy rhetoric into sustained investment and measurable action, Romania can reposition ageing from a symbol of dependency to a driver of public-health resilience and social inclusion. This paradigm shift, grounded in equity and participation, represents both a national imperative and a contribution to the collective European vision of healthier, more inclusive cities for all generations.
